# Zwitterionic-to-cationic charge conversion polyprodrug nanomedicine for enhanced drug delivery

**DOI:** 10.7150/thno.47849

**Published:** 2020-05-17

**Authors:** Sheng Wang, Fuwu Zhang, Guocan Yu, Zhantong Wang, Orit Jacobson, Ying Ma, Rui Tian, Hongzhang Deng, Weijing Yang, Zhi-Yi Chen, Xiaoyuan Chen

**Affiliations:** 1 School of Life Sciences, Tianjin University and Tianjin Engineering Center of Micro-Nano Biomaterials and Detection-Treatment Technology, Tianjin 300072, China; 2 Department of Ultrasound Medicine, Laboratory of Ultrasound Molecular Imaging, The Third Affiliated Hospital of Guangzhou Medical University, The Liwan Hospital of the Third Affiliated Hospital of Guangzhou Medical University, Guangzhou, Guangdong 510000, China; 3 Laboratory of Molecular Imaging and Nanomedicine, National Institute of Biomedical Imaging and Bioengineering, National Institutes of Health, Bethesda, Maryland 20892, United States

**Keywords:** charge conversion, zwitterionic, polyprodrug, triggered drug release, nanomedicine

## Abstract

Zwitterionic surface modification is a promising strategy for nanomedicines to achieve prolonged circulation time and thus effective tumor accumulation. However, zwitterion modified nanoparticles suffer from reduced cellular internalization efficiency.

**Methods:** A polyprodrug-based nanomedicine with zwitterionic-to-cationic charge conversion ability (denoted as ZTC-NMs) was developed for enhanced chemotherapeutic drug delivery. The polyprodrug consists of pH-responsive poly(carboxybetaine)-like zwitterionic segment and glutathione-responsive camptothecin prodrug segment.

**Results:** The ZTC-NMs combine the advantages of zwitterionic surface and polyprodrug. Compared with conventional zwitterionic surface, the ZTC-NMs can respond to tumor microenvironment and realize ZTC surface charge conversion, thus improve cellular internalization efficiency of the nanomedicines.

**Conclusions:** This ZTC method offers a strategy to promote the drug delivery efficiency and therapeutic efficacy, which is promising for the development of cancer nanomedicines.

## Introduction

Chemotherapy, which exploits small molecule antitumor drugs, is an indispensable approach in the clinic. In recent years, a wide range of chemotherapeutic drugs with high anticancer activities, such as doxorubicin, camptothecin (CPT) and paclitaxel, have been developed for chemotherapy 1-4. Unfortunately, most of these drugs suffer from nonspecific biodistribution, which may cause poor bioavailability and severe side effects. Nanomedicines offer an opportunity to address the mentioned limitations due to their potential of achieving improved drug delivery through the enhanced permeability and retention (EPR) effect 5-13. Polyprodrug strategy, which conjugates drugs to the polymer chains through responsive linkers, has been reported for the development of nanomedicine-based drug delivery systems 14-21. Compared to conventional drug carriers using physical loading approach, polyprodrug strategy shows distinct advantages such as high drug loading content, high loading stability and controlled drug release 22-28. Thus, polyprodrug-based nanomedicine is a promising approach for the delivery of chemotherapeutic drugs.

The surface modification is a very important factor for nanomedicines to achieve satisfactory *in vivo* performance. Neutral “stealth” coating is often favored for surface modification because it can prolong circulation time of nanomedicines and thus realize effective tumor accumulation 29-34. Particularly, zwitterionic structures, such as sulfobetaine and carboxybetaine, have recently received considerable attention as non-interacting surfaces that can reduce nonspecific adsorption 35, 36. Nanoparticles with zwitterionic surfaces exhibit high colloidal stability, long blood circulation time and high biocompatibility, resulting in effective tumor accumulation through the EPR effect 37-41. Furthermore, it has been demonstrated that zwitterionic poly(carboxybetaine) (PCB) modification can overcome the barriers associated with the use of polyethylene glycol (PEG) by avoiding the accelerated blood clearance phenomenon 42, 43. In order to achieve optimal treatment effect, nanomedicines are expected to effectively enter cancer cells once they reach tumor tissue. Unfortunately, compared to positively charged nanoparticles, zwitterionic nanoparticles suffer from reduced cellular internalization efficiency due to the poor zwitterionic surface-cell interactions 44, 45. In recent years, stimuli-responsive charge conversion strategy has been reported to promote the cellular internalization efficiency of nanomedicines 46-49. Under certain stimulus, the nanomedicines can achieve surface charge conversion from neutral or negative to positive, resulting in improved nanomedicine-cell interaction. However, the combining of polyprodrug nanomedicine and stimuli-responsive PCB-like modification with zwitterionic-to-cationic (ZTC) charge conversion ability to realize enhanced drug delivery has not yet been reported to the best of our knowledge.

**Figure 1:**
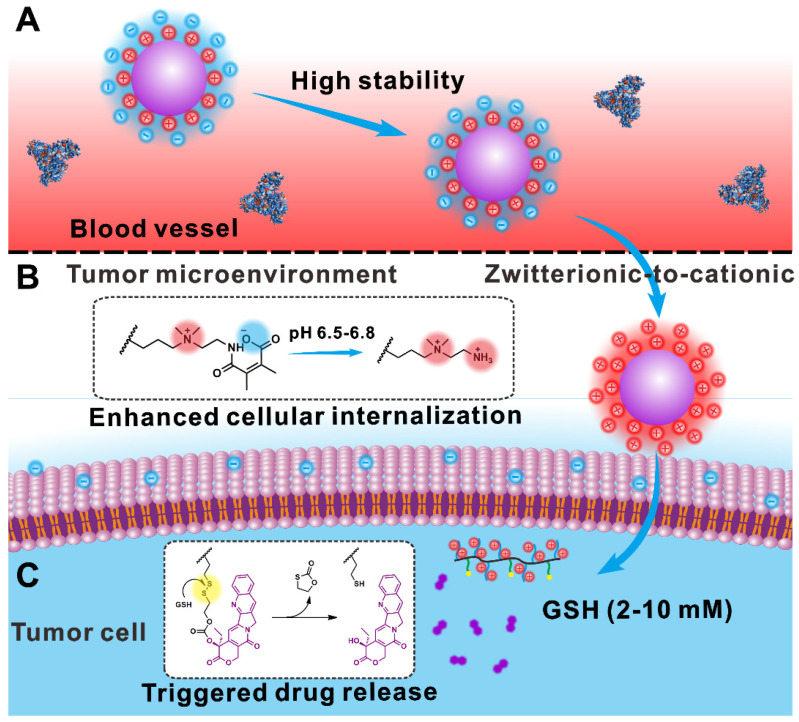
Schematic illustration showing the drug delivery process of ZTC-NMs. (A) The PCB-like zwitterion-modified nanomedicine shows high stability in blood circulation, resulting in efficient tumor accumulation through the EPR effect. (B) In tumor acidic microenvironment, the acid-induced zwitterionic-to-cationic surface charge conversion leads to enhanced cellular internalization of the nanomedicine. (C) The high level of intracellular GSH permits triggered drug release.

Herein, we report a pH-responsive PCB-like zwitterion-modified nanomedicine with ZTC charge conversion ability (denoted as ZTC-NM) for chemotherapeutic drug delivery. A polyprodrug that is composed of pH-responsive PCB-like zwitterionic segment and glutathione (GSH)-responsive poly-CPT segment was synthesized. The PCB-like zwitterionic structure consists of positively charged quaternary ammonium salt and negatively charged carboxyl group formed by the reaction of amino group and 2,3-dimethylmaleic anhydride (DMMA). Then the ZTC-NMs, which combined the advantages of polyprodrug and PCB-like modification, were prepared by self-assembly of the polyprodrug. As shown in Figure 1A, due to nanosized diameter and PCB-like zwitterionic surface modification, the ZTC-NMs can realize prolonged blood circulation time, which will facilitate tumor accumulation through the EPR effect. In tumor microenvironment, the amide bond formed between DMMA and amino group will respond to tumor pH (6.5-6.8) and achieve acid-responsive cleavage, leading to ZTC surface charge conversion of the ZTC-NMs 50. Due to the presence of highly positive quaternary ammonium salt, the resulting strong cationic NMs can rapidly enter tumor cells through effective electrostatic interaction with negatively charged cell membrane. Therefore, the ZTC charge conversion property can improve the cellular internalization efficiency of NMs and thus promote the drug delivery efficiency (Figure 1B). Moreover, after being taken up by tumor cells, the disulfide bonds in ZTC-NMs will be rapidly cleaved in the presence of intracellular GSH (Figure 1C), which would achieve triggered CPT release through a cascade reaction 51, 52.

## Results and Discussion

### Synthesis and characterization of polyprodrug

**Scheme 1:**
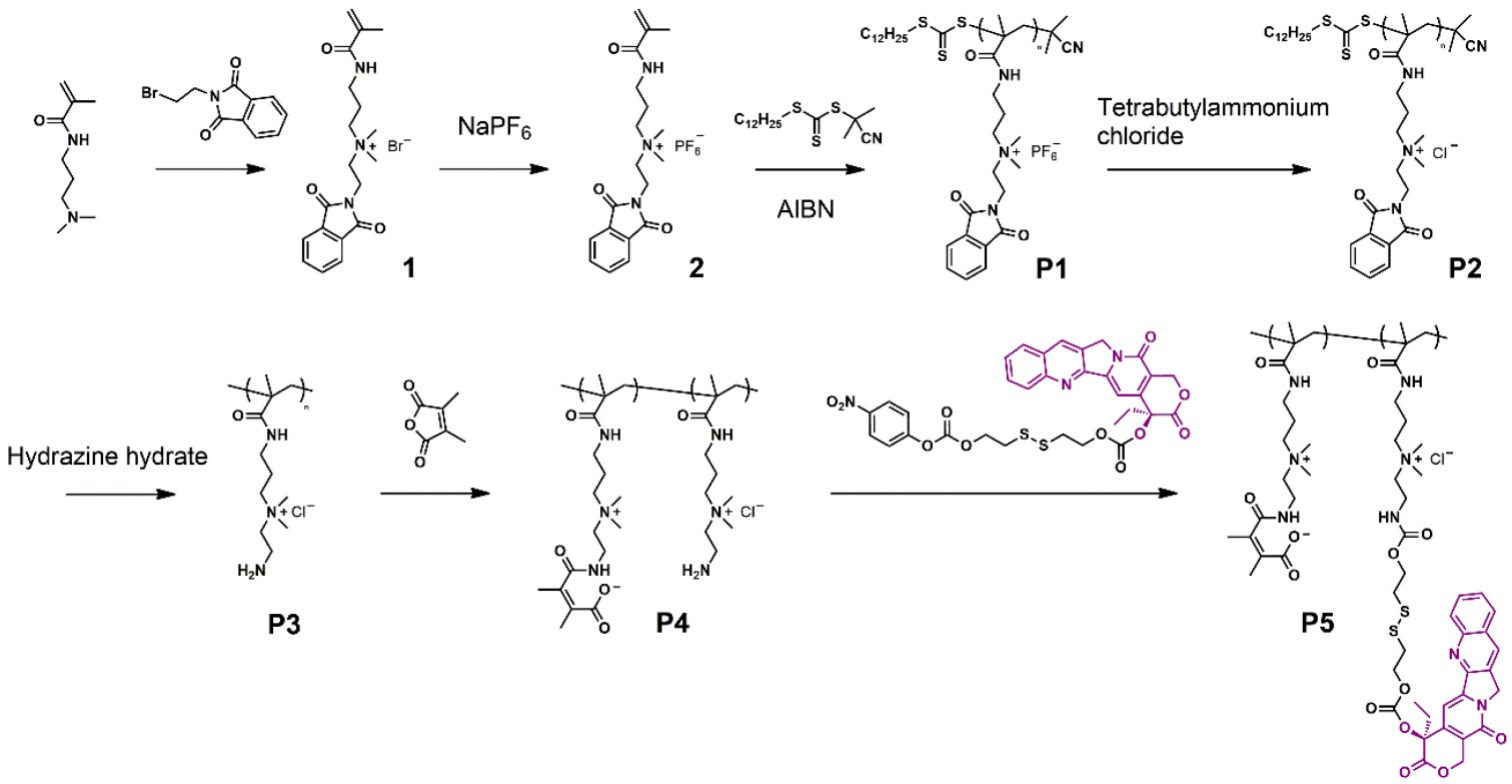
Synthesis process of the polyprodrug.

The dual-responsive polyprodrug (**P5**) was synthesized as shown in Scheme 1 and Scheme S1-S4. At first, a quaternary ammonium salt monomer (**1**) was synthesized by the reaction between N-[3-(dimethylamino)propyl]methacrylamide and N-(2-bromoethyl)phthalimide. Then the hexafluorophosphate salt monomer (**2**) was prepared for polymerization. Through reversible addition-fragmentation chain transfer (RAFT) polymerization, the quaternary ammonium salt polymer (**P1**) was synthesized. Afterwards, the **P1** was reacted with tetrabutylammonium chloride for ion exchange, obtaining **P2**. According to the nuclear magnetic resonance (NMR) results, the degree of polymerization and molecular weight of **P2** were determined to be 32 and 12.2 kDa, respectively (Figure S7). The synthesized **P2** was further treated with hydrazine hydrate to remove the protecting groups, yielding water-soluable polymer **P3**. Then DMMA was conjugated to the amino groups of **P3** to prepare the polymer with zwitterionic structure (**P4**). The **P5** was finally obtained by conjugating CPT to the zwitterionic polymer **P4** through GSH-responsive disulfide linker. To serve as a control group, a polyprodrug without pH-responsiveness (**P7**) was synthesized by the similar method, except that DMMA was replaced with succinic anhydride (Scheme S5). NMR, liquid chromatography-mass spectrometry (LC-MS) and UV-vis absorbance characterizations demonstrated the successful synthesis of monomers and polymers (Figure S1-S11). As calculated from the results of NMR spectra, approximately 75% of **P4** and **P6** were successfully linked with DMMA and succinic anhydride (Figure S9 and S10). By measuring the absorbance at 370 nm, the CPT loading contents of **P5** and **P7** were determined to be 14.9% and 16.5%, respectively (Figure S11).

### Preparation and characterization of the polyprodrug-based nanomedicine

**Figure 2:**
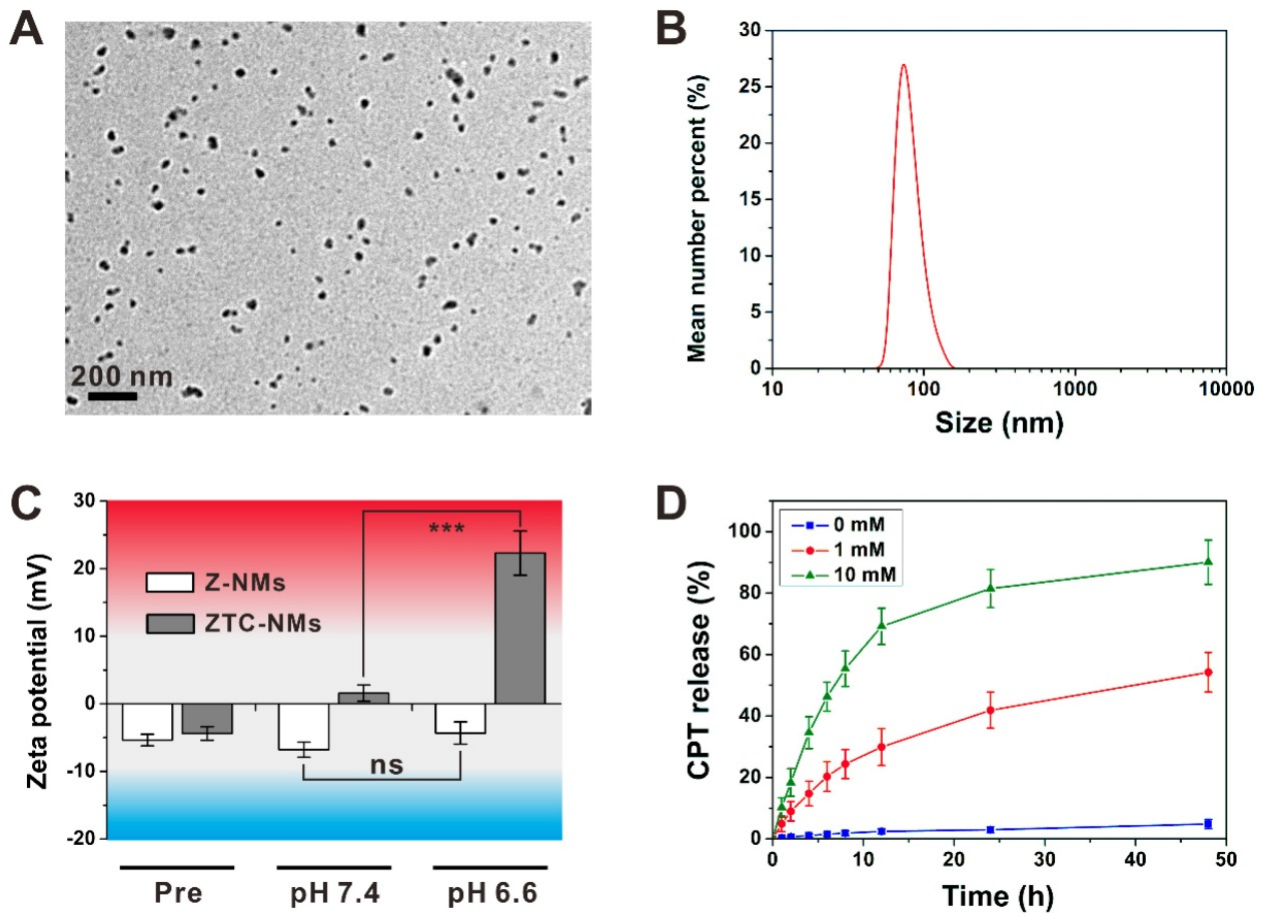
(A) Transmission electron microscope (TEM) image of ZTC-NMs. (B) Effective particle diameter of ZTC-NMs. (C) Zeta potential results of Z-NMs and ZTC-NMs after incubation at pH 7.4 and 6.6 for 3 h (*n* = 3, ***P < 0.001). (D) *In vitro* CPT release profiles of ZTC-NMs in the presence or absence of GSH (*n* = 3).

The amphiphilic polyprodrugs, **P5** and **P7**, can self-assemble into nanoparticles (ZTC-NMs and Z-NMs) with diameters of about 50 nm (Figure 2A and Figure S12). As shown in Figure 2B and Figure S13-S14, ZTC-NMs and Z-NMs showed similar hydrodynamic diameters and colloidal stabilities, which were characterized by dynamic light scattering (DLS) measurements. These results demonstrated that the prepared ZTC-NMs and Z-NMs were suitable for *in vivo* application. Then the pH-responsive property of the ZTC-NMs was determined by DLS. As shown in Figure 2C, both ZTC-NMs and Z-NMs showed neutral surface charges (between -10 mV and 10 mV) due to the zwitterionic surface modifications. After 3 h of incubation at pH 7.4, the ZTC-NMs still showed neutral surface charges. However, the surface charge of ZTC-NMs was converted into strong cationic (> 20 mV) after incubation at pH 6.6 for 3 h, which was due to the pH-induced detachment of DMMA groups and the presence of highly positive quaternary ammonium salt. In general, the nanomedicines that accumulate in tumor tissue through EPR effect can retain for a long time (more than 24 h); therefore, the ZTC-NMs that can achieve ZTC charge conversion within 3 h are suitable for tumor microenvironment-responsive drug delivery. In contrast, after incubation at different pH, the Z-NMs didn't show obvious change in surface charge due to the non-responsiveness of zwitterionic structure in Z-NMs. The size changes of ZTC-NMs in different conditions were also determined. As shown in Figure S15, the size of the ZTC-NMs didn't show obvious change at both pH 7.4 and pH 6.6. Then the GSH-responsive drug release behavior of the ZTC-NMs was investigated by dialysis method. In the absence of GSH, the ZTC-NMs didn't show obvious drug leakage (Figure 2D). Considering the low GSH concentration in body fluids and in the extracellular milieu, the relatively high stability of the nanomedicines led to minimal side effects. This is one of the important advantages of polyprodrug-based nanomedicines when compared with nanomedicines that based on physical encapsulation approach. However, when incubated with 1 × 10^-3^ M GSH, accelerated drug release was observed, with more than 50% CPT released in 48 h. The drug release rate was further increased when the ZTC-NMs were incubated with higher concentration GSH (1 × 10^-2^ M). To investigate the release mechanism of the ZTC-NMs in the presence of GSH, the release medium was analyzed by LC-MS. A peak at m/z 349.1 (calculated as 349.1) was found in the spectrum (Figure S16), indicating that free CPT was released. These results demonstrated that the GSH can trigger free CPT release by cleavage of disulfide bonds and subsequent cascade reaction 51-54. It has been demonstrated that the GSH concentration inside cancer cells is relatively high; therefore, the ZTC-NMs would rapidly release CPT inside cancer cells.

### *In vitro* cellular study

**Figure 3:**
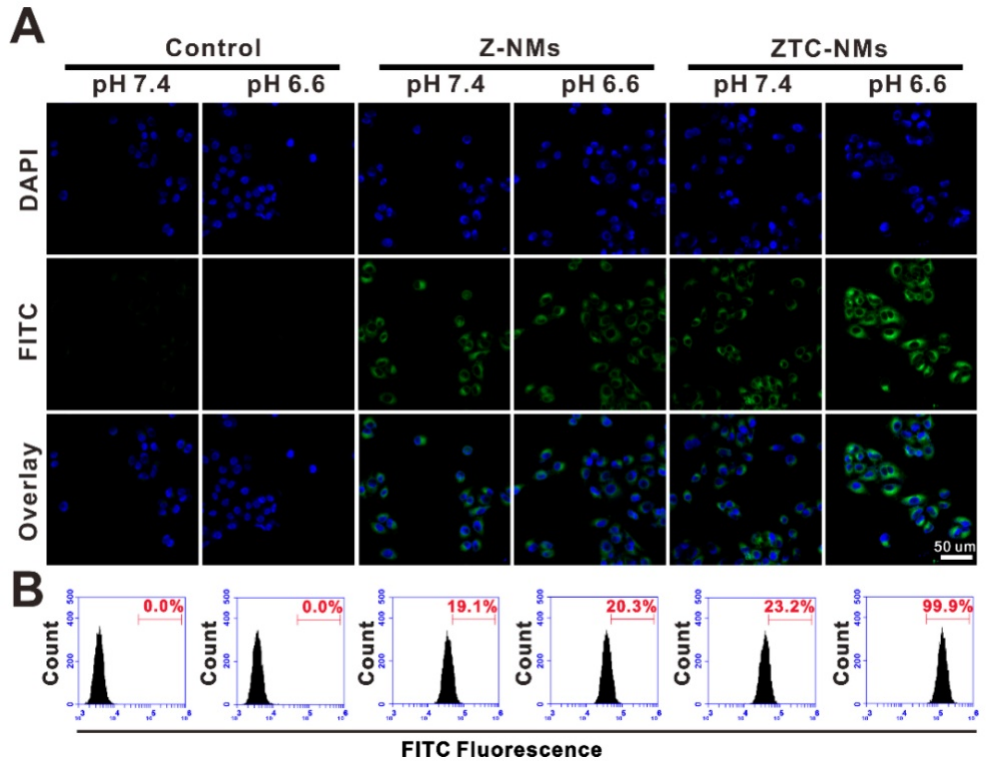
(A) Confocal fluorescence images and (B) flow cytometry (FCM) analysis of A549 cells upon incubation with different pH pretreated FITC-labeled Z-NMs and ZTC-NMs for 2 h.

**Figure 4:**
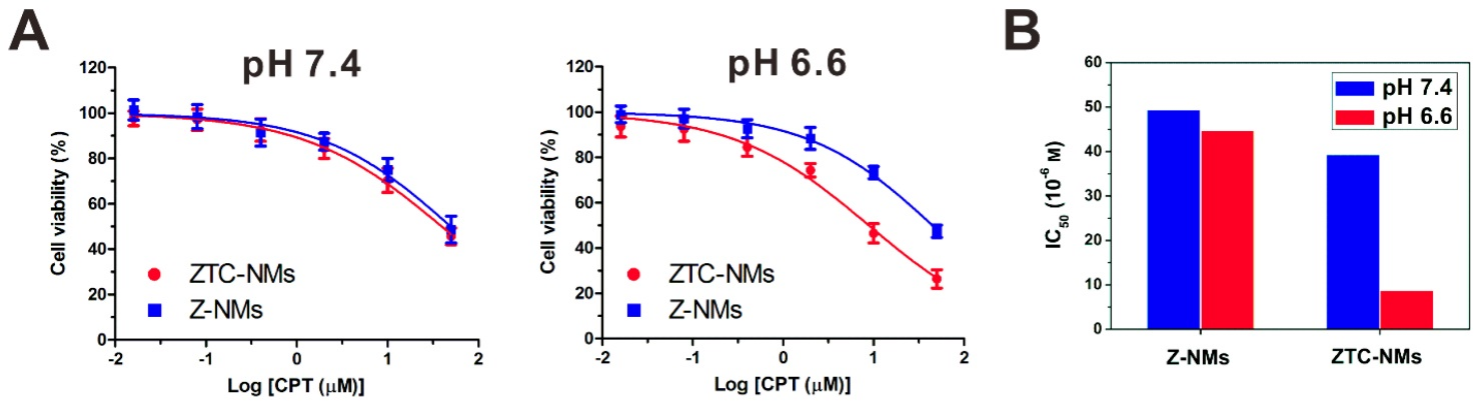
(A) Viability of A549 cells incubated with ZTC-NMs and Z-NMs at different pH for 6 h and then incubated with fresh culture medium for another 42 h. (*n* = 5/group) (B) The IC_50_ values of Z-NMs and ZTC-NMs at different pH (based on data in (A)).

The enhanced cellular internalization of the ZTC-NMs was evaluated on A549 cells. To observe the cellular uptake of nanomedicines, both ZTC-NMs and Z-NMs were labeled by fluorescein isothiocyanate (FITC). The FITC-labeled NMs were pretreated with pH 7.4 or 6.6 buffer solution for 3 h. Then A549 cells were incubated with pretreated ZTC-NMs or Z-NMs for 2 h. Thereafter, cells were stained with 4',6-diamidino-2-phenylindole (DAPI) and analyzed by confocal laser scanning microscopy (CLSM) and flow cytometry (FCM). As shown in Figure 3A and 3B, cells with pH 7.4 pretreated ZTC-NMs incubation showed relatively weak fluorescence. The low cellular uptake may be attributed to the zwitterionic surface of the ZTC-NMs. However, obviously stronger green fluorescence was found inside the cells when incubated with pH 6.6 pretreated ZTC-NMs, indicating enhanced cellular internalization of the ZTC-NMs. In contrast, both pH 7.4 and pH 6.6 pretreated Z-NMs showed low cellular uptake, indicating non-responsiveness of the Z-NMs. These results confirmed that the pH-responsive ZTC charge conversion ability of the ZTC-NMs could enhance intracellular drug delivery in weak acidic environment. Then the cell growth inhibition efficacies of ZTC-NMs and Z-NMs were further evaluated. Cells were incubated with pH 7.4 and pH 6.6 pretreated NMs for 6 h and then with fresh culture medium for additional 42 h. Next, a methyl thiazolyl tetrazolium (MTT) assay was conducted to measure the cell viabilities. As shown in Figure 4A, the pH 7.4 pretreated ZTC-NMs and Z-NMs showed comparable cell growth inhibition ability; however, compared to Z-NMs, the pH 6.6 pretreated ZTC-NMs showed much improved cytotoxicity. The calculated IC_50_ value of pH 6.6 pretreated ZTC-NMs was 5.1-fold lower than that of pH 6.6 pretreated Z-NMs (Figure 4B). This pH-induced high anticancer efficacy can be a result of ZTC charge conversion-enhanced intracellular drug delivery.

### *In vivo* positron emission tomography (PET) imaging

**Figure 5:**
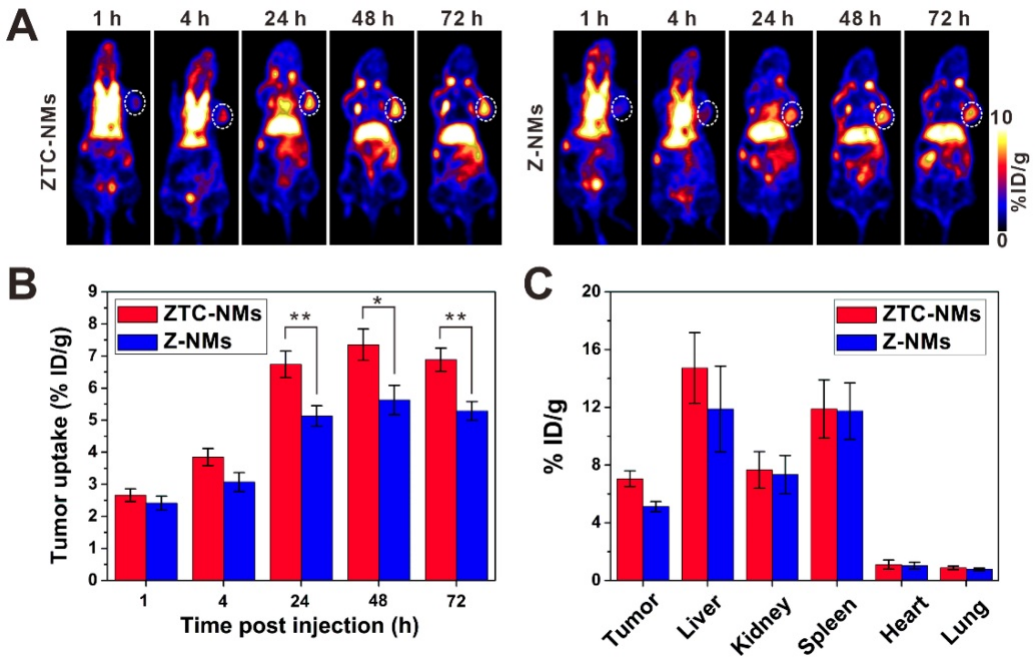
(A) PET images of A549 tumor-bearing mice after intravenous injection of ^89^Zr-Z-NMs or ^89^Zr-ZTC-NMs. The white circles indicate the tumor area. (B) Tumor uptake efficiencies of the ^89^Zr-Z-NMs or ^89^Zr-ZTC-NMs at different time points. (C) Biodistribution of tumor and primary organs at 72 h postinjection. (*n* = 3/group, *P < 0.05, **P < 0.01).

Next, the *in vivo* performance of ZTC-NMs was studied by PET imaging. Zirconium-89 (^89^Zr) was used for radiolabeling. The ^89^Zr-Z-NMs and ^89^Zr-ZTC-NMs were injected intravenously into A549 tumor-bearing mice for PET image acquisition at 1, 4, 24, 48 and 72 h postinjection. Quantitative analysis in heart (with blood) region demonstrated long blood circulation times of the ZTC-NMs and Z-NMs (Figure S17). The acquired PET images at different time points indicated that both ZTC-NMs and Z-NMs effectively accumulated in the tumor region, probably due to the EPR effect of nanomedicines (Figure 5A). The long retention time facilitates the effective response of the ZTC-NMs to tumor microenvironment stimulation and realize ZTC charge conversion. Compared with Z-NMs, ZTC-NMs showed obviously higher tumor accumulation and retention at 24, 48 and 72 h postinjection (Figure 5B). Considering the similar size and surface property of ZTC-NMs and Z-NMs, the enhanced tumor accumulation and retention of ZTC-NMs can be a result of high cell-nanomedicine interaction, resulting from tumor microenvironment-induced ZTC charge conversion. At 72 h postinjection, *ex vivo* biodistribution study was performed by quantifying radioactivity in the tumor and different organs using a γ-counter (Figure 5C). The result was consistent with quantitative PET region-of-interest analysis, indicating enhanced drug delivery ability of the ZTC-NMs.

### *In vivo* antitumor study

**Figure 6:**
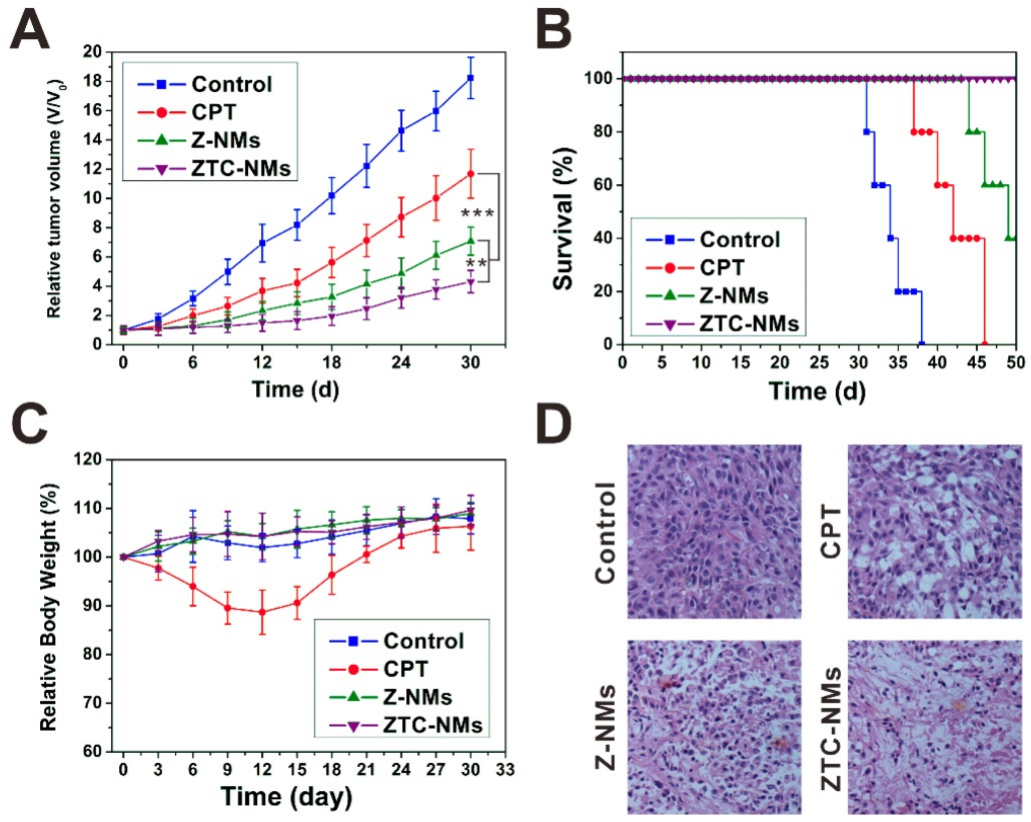
(A) Tumor growth curves of the A549 tumor-bearing mice treated with different formulas. (B) Survival curves of the mice treated with different samples. (C) Mouse body-weight changes during the treatments. (D) H&E analyses of tumor tissues after different treatments. (**P < 0.01, ***P < 0.001).

Encouraged by the effective tumor targeting behavior, the *in vivo* antitumor effect of ZTC-NMs was further evaluated on A549 tumor mice. Free CPT was used as a control. Saline or different formulations were intravenously injected into tumor-bearing mice every 3 days for 5 times (CPT equivalent dose: 3 mg kg^-1^). As shown in Figure 6A, both free CPT and nanomedicine treatments delayed tumor growth. The free CPT displayed only moderate antitumor effect; however, the NMs showed more effective tumor inhibition, which could be attributed to the tumor targeted drug delivery. Importantly, compared with Z-NMs, the ZTC-NMs showed the most potent antitumor effect (Figure 6D and Figure S18). As a result, the survival time of ZTC-NM-treated mice was greatly prolonged (Figure 6B). This result demonstrated that the ZTC charge conversion ability was conducive to enhance drug delivery. Meanwhile, during the treatment period, the mice treated with ZTC-NMs and Z-NMs didn't show obvious body weight loss or organ damages; however, free CPT-treated mice suffered from systemic toxicity induced weight loss (Figure 6C and Figure S19). These results indicated that the NMs reduced side effects caused by anticancer drug.

## Conclusions

In summary, a nanomedicine with zwitterionic-to-cationic surface charge conversion ability was developed for enhanced delivery of chemotherapeutic drug. The nanomedicine was prepared by self-assembly of amphiphilic polymer which contains pH-responsive PCB-like zwitterionic segment and GSH-responsive prodrug segment. Owing to the zwitterionic surface modification, the nanomedicine exhibited high stability and prolonged circulation time. Furthermore, the ZTC charge conversion ability of the nanomedicine in tumor microenvironment promoted cell internalization, which resulted in enhanced tumor accumulation and retention. In tumor cells, controlled drug release was achieved through GSH-triggered cleavage of disulfide bonds. Considering the effective tumor inhibition and low systemic toxicity *in vivo*, the ZTC-NMs have the potential for anticancer drug delivery.

## Methods

### Preparation and characterizations of nanomedicines

Briefly, **P5** (1 mg) was dissolved in 500 μL of DMSO and then added into 4 mL of distilled water dropwise under stirring. The organic solvents were removed through ultrafiltration, obtaining ZTC-NMs. The Z-NMs were prepared under the same experimental conditions except that the **P5** was replaced with **P7**. The morphologies, effective particle diameters and Zeta potentials of the samples were studied by Tecnai TF30 TEM (FEI, Hillsboro) and SZ-100 nanoparticle analyzer (HORIBA Scientific). UV-vis absorption spectra of the samples were measured by Genesys 10S UV-Vis spectrophotometer (Thermo Scientific).

### *In vitro* drug release

The *in vitro* GSH-triggered CPT release behaviors of the samples were evaluated by using a dialysis method. The ZTC-NMs were dispersed in 2 mL of media (phosphate buffered saline, 1 mM GSH or 10 mM GSH) and added to dialysis bags (MWCO: 3500 Da). Then the dialysis bags were placed in 20 mL of environmental media (*n* = 3) and incubated at 37 °C. At appropriate time points, 2 mL of the environmental medium was taken out for quantification of released CPT by high performance liquid chromatography at the wavelength of 370 nm. And same amount of fresh medium was added. To verify that the free CPT molecules were released by cleavage of disulfide bonds and subsequent cascade reaction, the release medium was analyzed by using LC-MS.

### *In vitro* cell experiments

The *in vitro* cellular uptake study and antitumor activity study were assessed on A549 cell line, which was purchased from American type culture collection (ATCC). To investigate the cellular uptake of ZTC-NMs and Z-NMs, A549 cells were seeded into 8-well plates at a density of 2 × 10^4^ cells per well and incubated at 37 °C for 24 h. Then, pretreated (at pH 7.4 or 6.6 for 3 h) FITC-labeled NMs were added to wells and incubated for 2 h. Then the culture media were removed, and the cells were fixed with Z-Fix solution and stained with DAPI. Then the cellular uptake was determined by confocal images and flow cytometry analyses. For *in vitro* cell experiments, A549 cells were seeded into 96-well plates at a density of 3 × 10^3^ cells per well and incubated with different formulas at different pH for 6 h to allow ZTC charge conversion and cellular internalization (*n* = 5/group). Then the culture medium was replaced with fresh medium and the cells were incubated for another 42 h. Thereafter, the relative cell viabilities were measured by MTT assay. The IC_50_ values were calculated by using GraphPad Prism 5.

### *In vivo* PET imaging

All animal experiments were performed under a National Institutes of Health Animal Care and Use Committee (NIHACUC) approved protocol. Athymic nude mice (Harlan, Indianapolis, IN) were subcutaneously implanted with 3 × 10^6^ A549 cells. Deferoxamine (DFO) conjugated polyprodrug was synthesized for ^89^Zr labeling (Scheme S6) 24, 55. The ^89^Zr-ZTC-NMs or ^89^Zr-Z-NMs solution (100 μL, 200 μCi) was intravenously injected into A549 tumor-bearing mice (*n* = 3/group). At appropriate time points after injection, PET images were acquired by using an Inveon small-animal PET scanner (Siemens, Erlangen, Germany). At 72 h post-injection, the mice were sacrificed for biodistribution study. Tumors and major organs were collected and assayed for radioactivity using a gamma counter. The percent injected dose/gram of tissue (%ID/g) was then calculated.

### *In vivo* therapy

A549 tumor-bearing mice were randomly divided into 4 groups (*n* = 6/group): control group, free CPT group, ZTC-NMs group and Z-NMs group. When the tumors reached 80 mm^3^, the mice were treated with different formulas (3 mg CPT kg^-1^ equivalent) *via* intravenous injection every 3 days for 5 times. Tumor volume and body weight were monitored every 3 days. Tumor volume was calculated as (major axis) × (minor axis)^2^/2. Based on the Animal Study Protocol, mice with oversized tumor (major axis > 20 mm) or significant weight loss (> 20%) were euthanized. After treatment, one mouse from each group was euthanized, major organs and tumors were collected for terminal deoxynucleotidyl transferase dUTP nick end labeling (TUNEL) assay and hematoxylin and eosin (H&E) staining 56.

## Abbreviations

CPT: camptothecin
EPR: enhanced permeability and retention
PCB: poly(carboxybetaine)
PEG: polyethylene glycol
ZTC: zwitterionic-to-cationic
NM: nanomedicine
GSH: glutathione
DMMA: 2,3-dimethylmaleic anhydride
RAFT: reversible addition-fragmentation chain transfer
NMR: nuclear magnetic resonance
LC-MS: liquid chromatography-mass spectrometry
DLS: dynamic light scattering
TEM: transmission electron microscope
FITC: fluorescein isothiocyanate
DAPI: 4',6-diamidino-2-phenylindole
CLSM: confocal laser scanning microscopy
FCM: flow cytometry
MTT: methyl thiazolyl tetrazolium
PET: positron emission tomography
^89^Zr: Zirconium-89
DFO: deferoxamine
TUNEL: terminal deoxynucleotidyl transferase dUTP nick end labeling
H&E: hematoxylin and eosin
