# Supplementary material for: Zwitterionic-to-cationic charge conversion polyprodrug nanomedicine for enhanced drug delivery
Source: Theranostics. 2020 May 17;10(15):6629–37. doi: 10.7150/thno.47849 (PMC7295052; doi:10.7150/thno.47849)
Supplement: Supplementary file 1 — Supplementary figures and tables. [file thnov10p6629s1.pdf]

Supporting Information

Zwitterionic-to-cationic charge conversion polyprodrug nanomedicine for enhanced drug delivery

Sheng Wang, Fuwu Zhang, Guocan Yu, Zhantong Wang, Orit Jacobson, Ying Ma, Rui Tian, Hongzhang Deng, Weijing Yang, Zhi-Yi Chen*, and Xiaoyuan Chen**

Materials

2-Cyano-2-propyl dodecyl trithiocarbonate, 2-hydroxyethyl disulfide, N-[3-(dimethylamino)propyl] methacrylamide (DMAPMA), N-(2-bromoethyl) phthalimide, 4-nitrophenyl chloroformate, sodium hexafluorophosphate, tetrabutylammonium chloride, 2,3-dimethylmaleic anhydride (DMMA), succinic anhydride (SA), 2,2′-azobis(2-methylpropionitrile) (AIBN), methyl thiazolyl tetrazolium (MTT), 4′,6-diamidino-2-phenylindole (DAPI) were obtained from Sigma-Aldrich. Hydrazine hydrate was purchased from TCI. (S)-(+)-camptothecin (CPT) was purchased from Chem-Impex (USA). p-SCN-Bn-Deferoxamine (SCN-DFO) was purchased from Macrocyclics.

Synthetic Methods

Synthesis of 1 and 2

The **1** and **2** were synthesized as shown in Scheme S1. DMAPMA (10 mmol) and N-(2-bromoethyl)phthalimide (10 mmol) were dissolved in 5 mL of acetonitrile. Then the mixture was stirred at 60 °C for 48 h. The product was purified by precipitating into 100 mL of diethyl ether. The precipitate was washed twice with diethyl ether and then dried under vacuum. Then the **2** was synthesized as follows: **1** (10 mmol) was dissolved in 40 mL of pure water. Then sodium hexafluorophosphate was added into the solution to precipitate the **2**. The precipitate was washed twice with pure water and then lyophilized.

Synthesis of 3 and 4

The **3** and **4** were synthesized as shown in Scheme S2. The **3** was first synthesized according previous report.[1] The **3** (1 mmol) and triethylamine (1.2 mmol) were dissolved in 20 mL of dichloromethane. Then 4-nitrophenyl chloroformate (1.2 mmol) was added into the above mixture at 0 °C. The reaction was carried out for 24 h at room temperature. The mixture was washed twice with water and brine. The organic layer was separated and dried over anhydrous $MgSO_4$. The **4** was purified by precipitating into diethyl ether.

Synthesis of P1, P2 and P3

The **P1** was first synthesized by a Reversible Addition–Fragmentation chain Transfer (RAFT) polymerization method as shown in Scheme S3. Briefly, 2-cyano-2-propyl dodecyl trithiocarbonate (0.05 mmol), **2** (3 mmol), AIBN (0.0125 mmol) were dissolved in 4 mL of DMF and added into a flask. The flask was sealed under dry argon and the polymerization was carried out at 80 °C for 24 h. Then 10 mL of acetone was added into the **P1** solution. The obtained mixture was added into 30 mL of tetrabutylammonium chloride (1 g) acetone solution. The precipitate was washed twice with acetone and then dialyzed (MWCO: 1000 Da) against pure water. Finally, the solution was lyophilized to generate **P2**. The synthesized **P2** was dissolved in 10 mL of ethanol. Hydrazine hydrate (2 mL) was added to the solution and the solution was heated at reflux for 6 h and then cooled to room temperature. The mixture was dialyzed (MWCO: 1000 Da) against pure water and then lyophilized to generate **P3**.

Synthesis of P4

The **P4** was synthesized as shown in Scheme S4. In brief, **P3** (250 mg) was dissloved in 5 mL of DMSO. Then triethylamine (150 μL), pyridine (150 μL) and DMMA (126 mg) were added into the **P4** solution under N_2 atmosphere. The mixture was stirred at room temperature for 24 h. The resulting solution was dialyzed (MWCO: 1000 Da) against pure water for 24 h and then lyophilized to generate **P4**.

Synthesis of P5

The **P4** was dissolved in 5 mL of DMF. Then triethylamine (160 μL) and **4** (208 mg) were added into the solution. The mixture was stirred at room temperature for 24 h. The resulting solution was dialyzed (MWCO: 1000 Da) against DMF for 24 h and then dried under vacuum.

Synthesis of P6 and P7

The **P6** and **P7** were synthesized under the same experimental conditions except that the DMMA was replaced with SA (Scheme S5).

Synthesis of DFO-P7

The **DFO-P7** was synthesized through the reaction between SCN-DFO and excessive **P6** in DMF. The obtained DFO-**P6** was further reacted with **4** by the above-mentioned method to synthesize **DFO-P7** (Scheme S6).

[1] Zhang F, Zhu G, Jacobson O, Liu Y, Chen K, Yu G, et al. Transformative nanomedicine of an amphiphilic camptothecin prodrug for long circulation and high tumor uptake in cancer therapy. ACS Nano. 2017; 11: 8838-48.

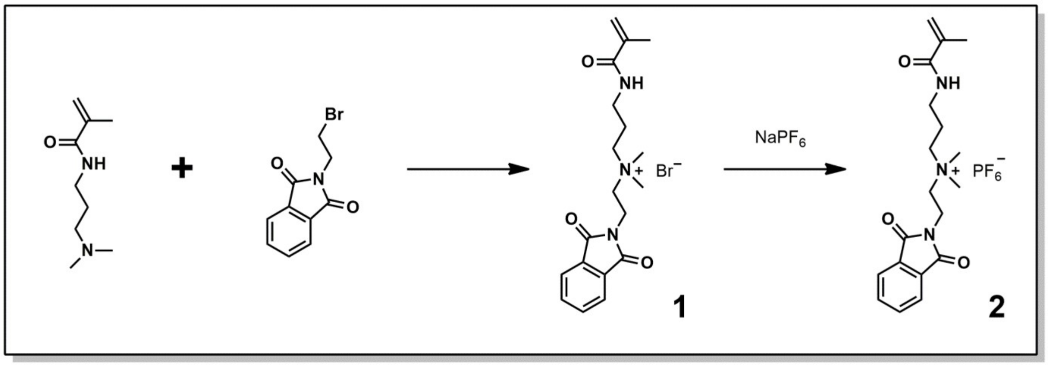

Scheme S1. Synthesis processes of **1** and **2**.

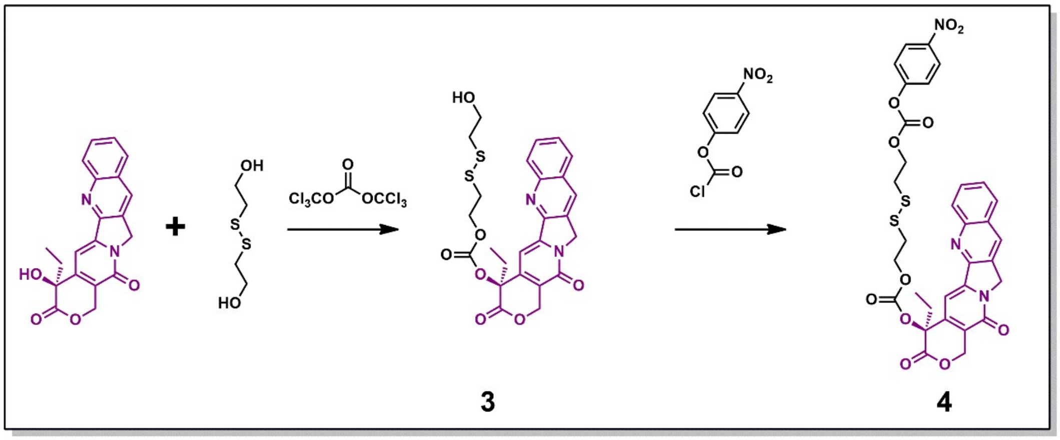

Scheme S2. Synthesis processes of **3** and **4**.

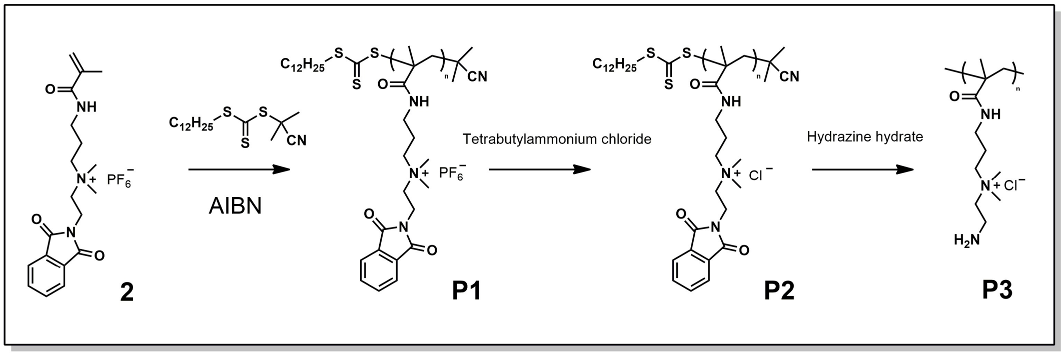

Scheme S3. Synthesis processes of **P1**, **P2** and **P3**.

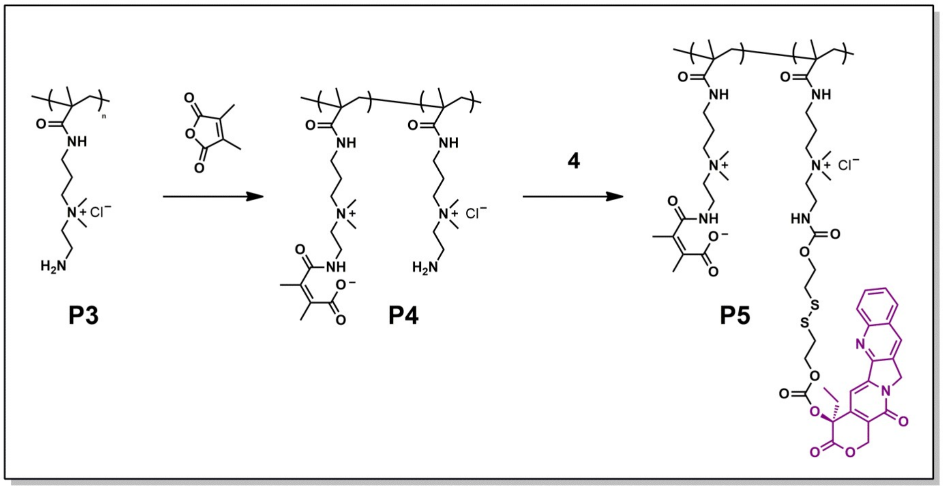

Scheme S4. Synthesis processes of **P4** and **P5**.

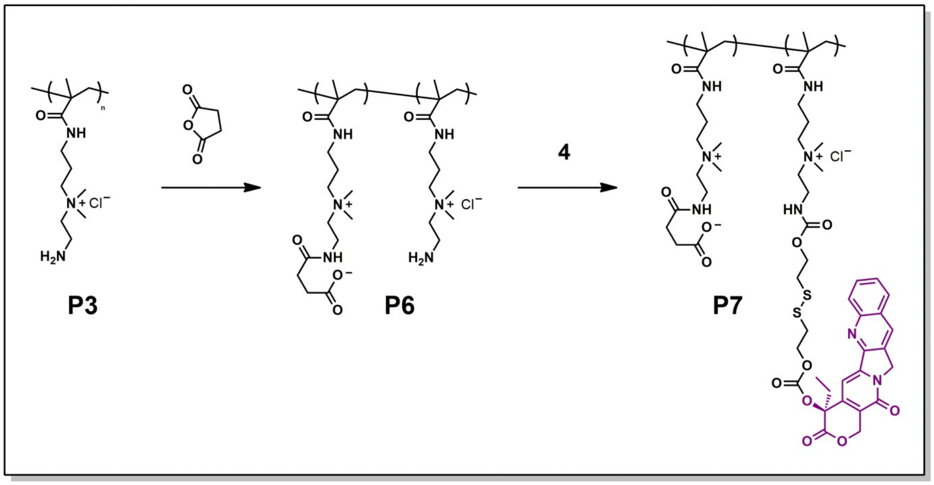

Scheme S5. Synthesis processes of **P6** and **P7**.

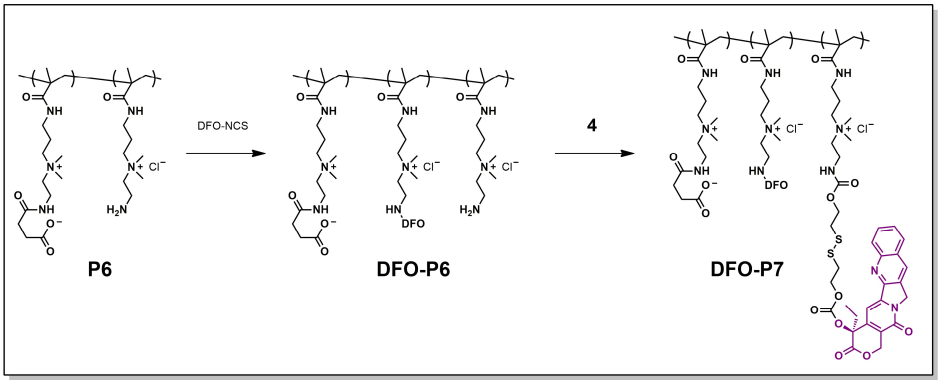

Scheme S6. Synthesis processes of DFO-**P7**.

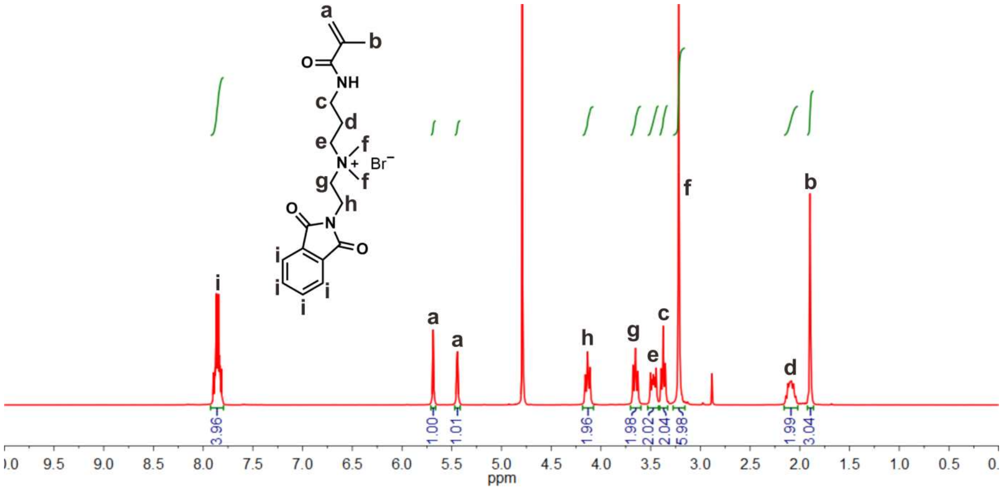

Figure S1. ^{1}H NMR spectrum of **1**.

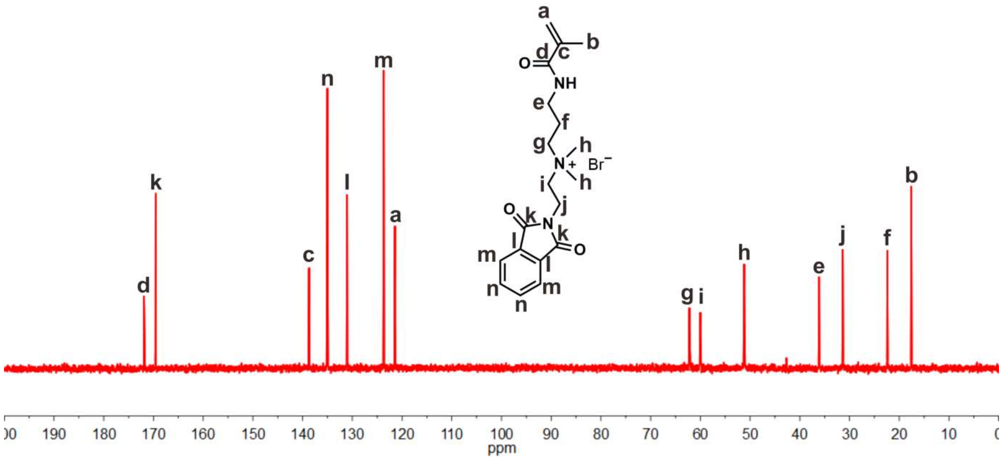

Figure S2. ^{13}C NMR spectrum of **1**.

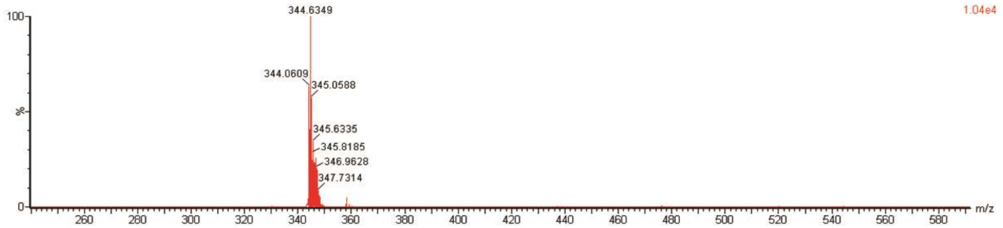

Figure S3. Mass spectrum of **1**.

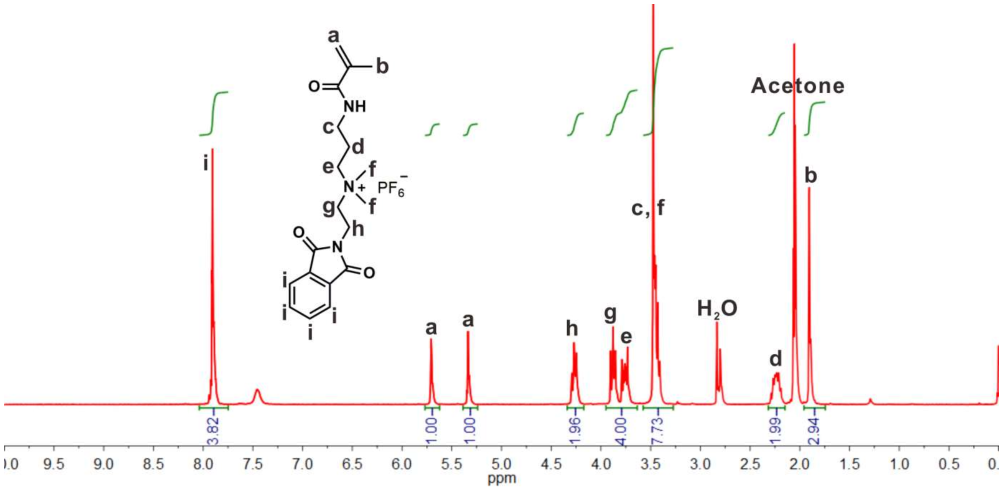

Figure S4. ^{1}H NMR spectrum of **2**.

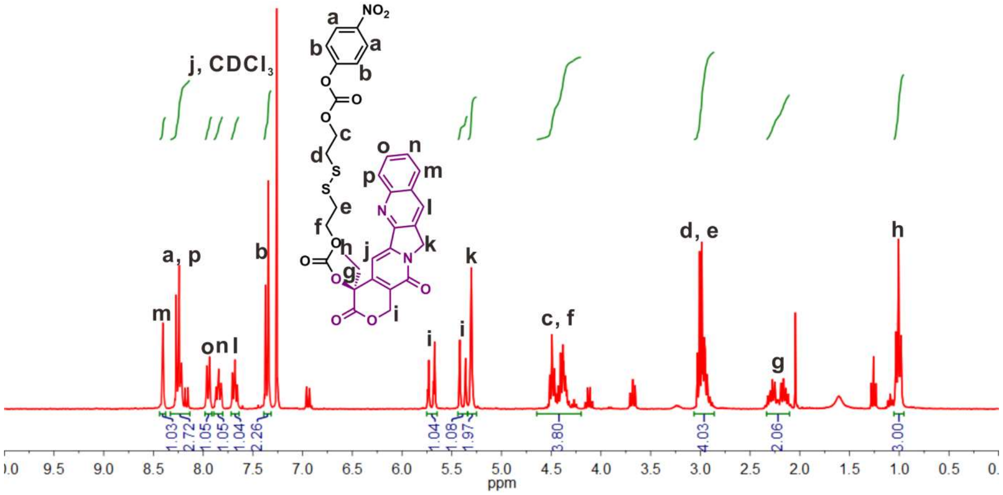

Figure S5. ^{1}H NMR spectrum of **4**.

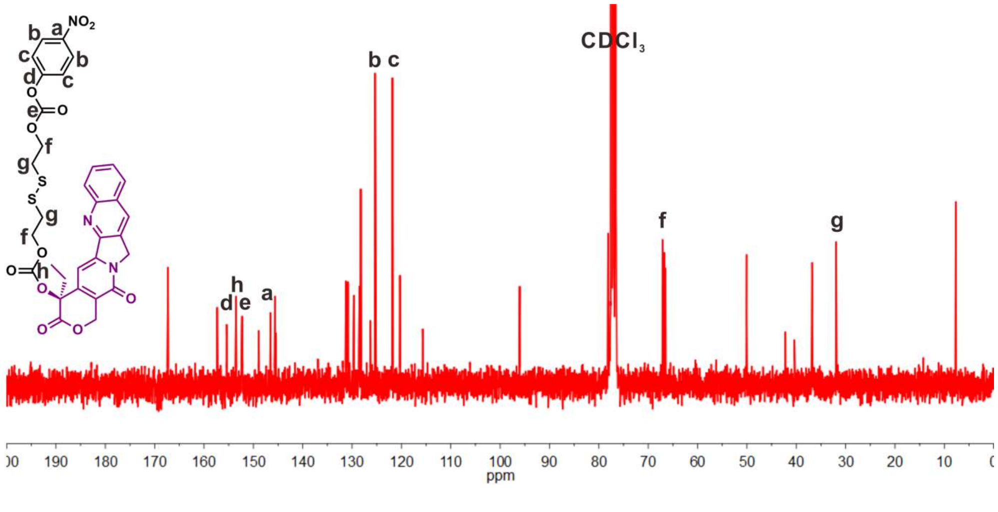

Figure S6. ^{13}C NMR spectrum of **4**.

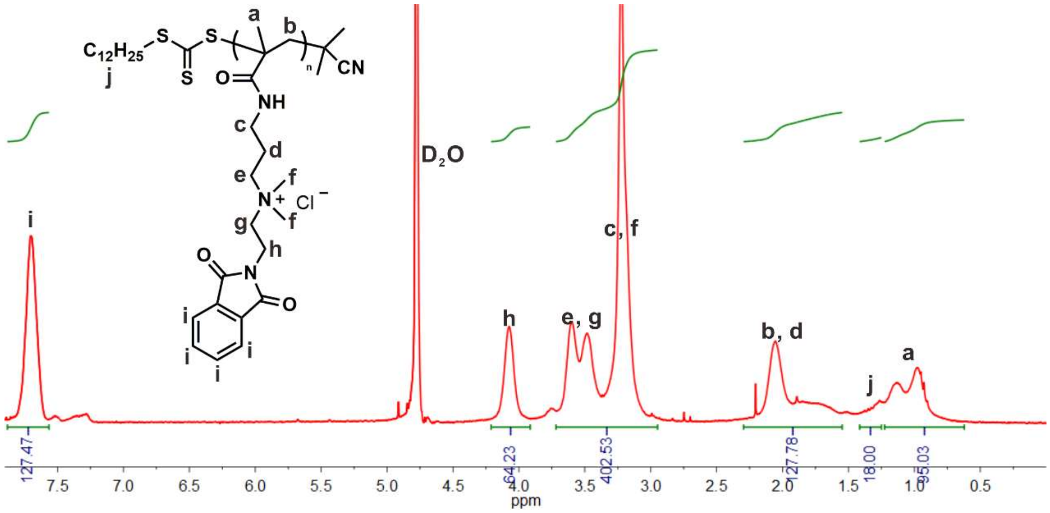

Figure S7. ^{1}H NMR spectrum of **P2**.

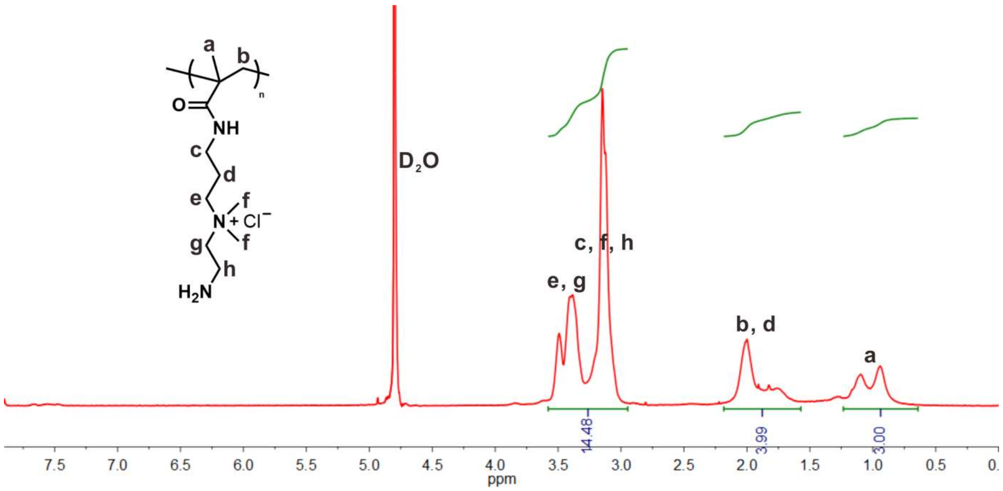

Figure S8. ^{1}H NMR spectrum of **P3**.

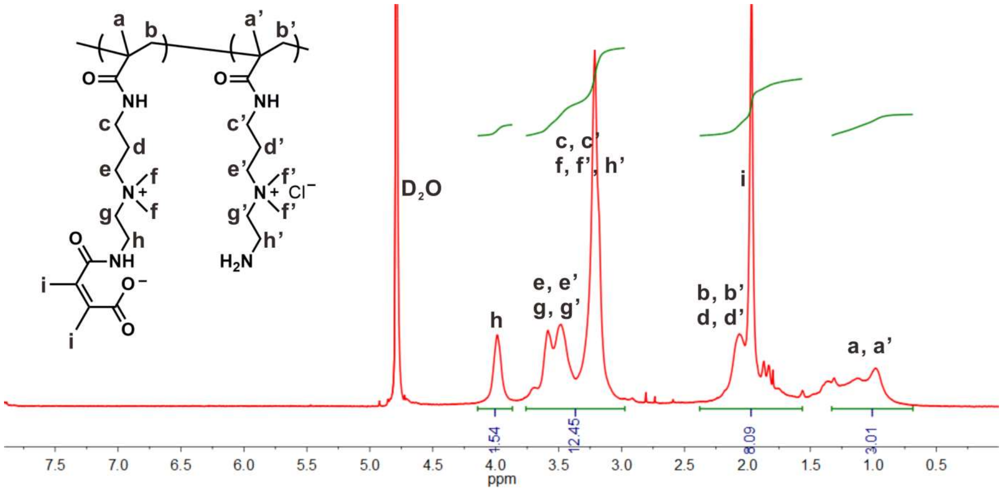

Figure S9. ^{1}H NMR spectrum of **P4**.

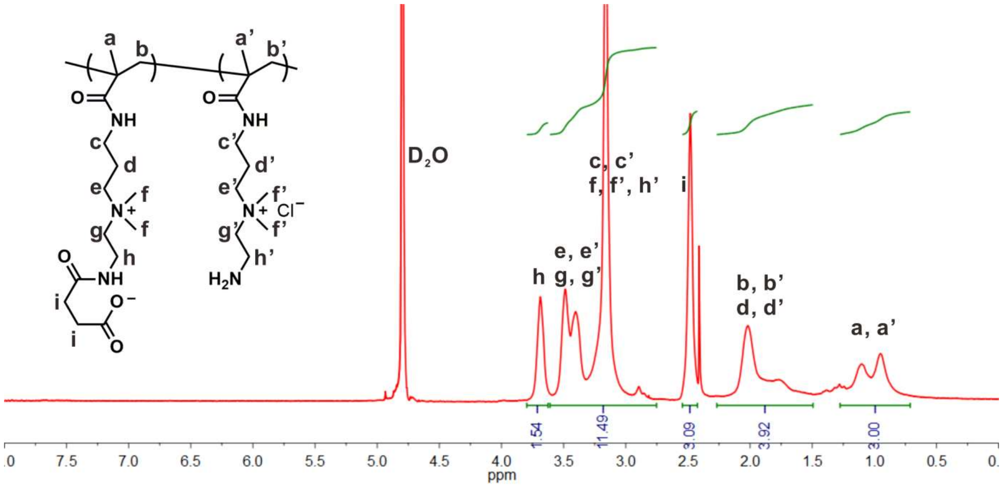

Figure S10. ^{1}H NMR spectrum of **P6**.

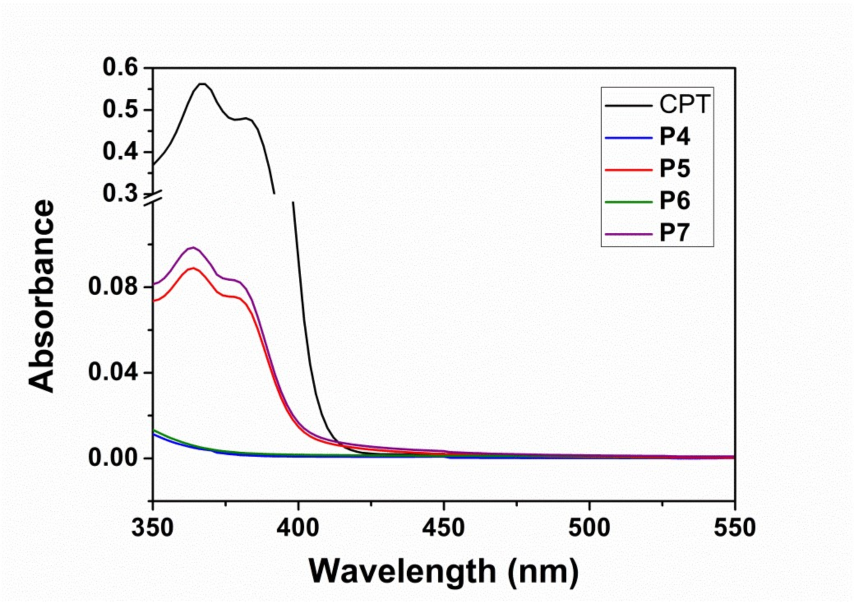

Figure S11. Absorption spectra of CPT, **P4**, **P5**, **P6** and **P7**.

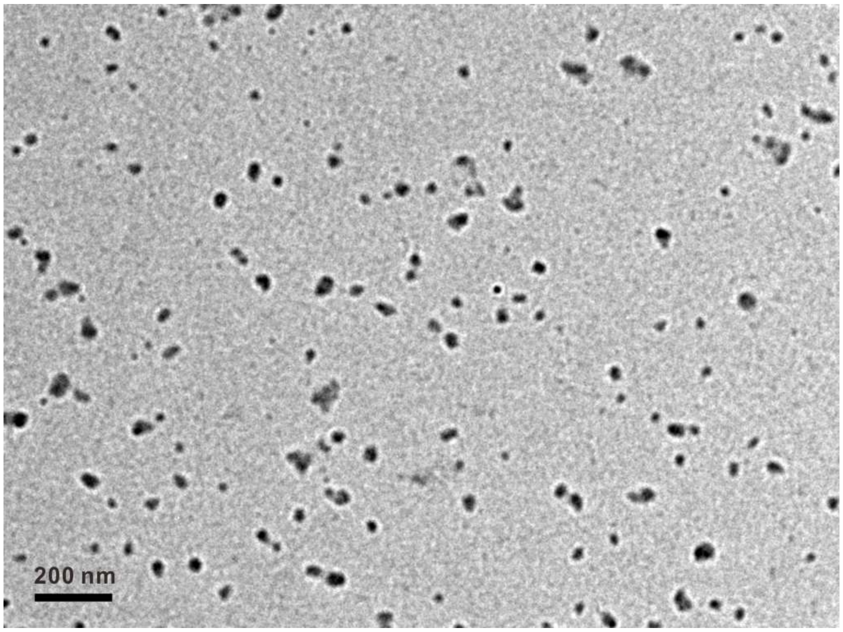

Figure S12. TEM image of Z-NMs.

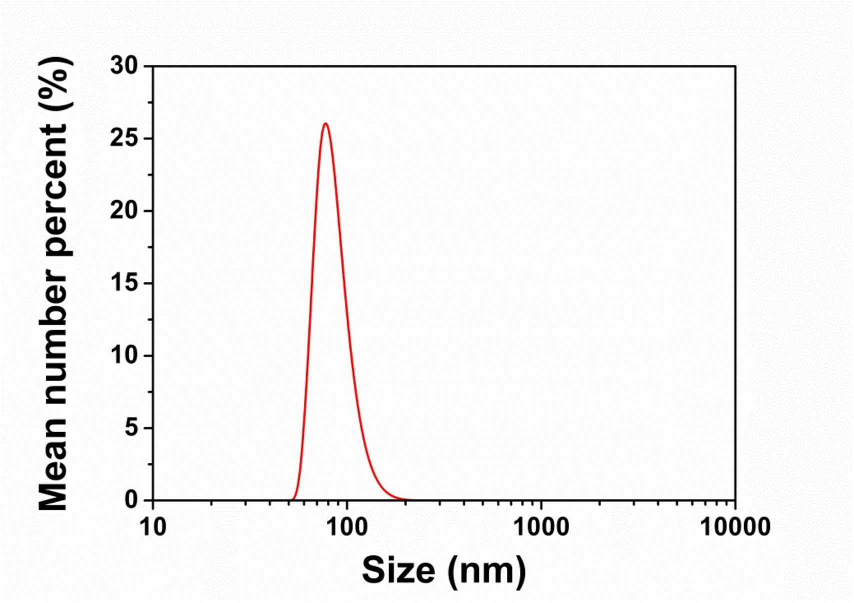

Figure S13. Particle diameter of Z-NMs.

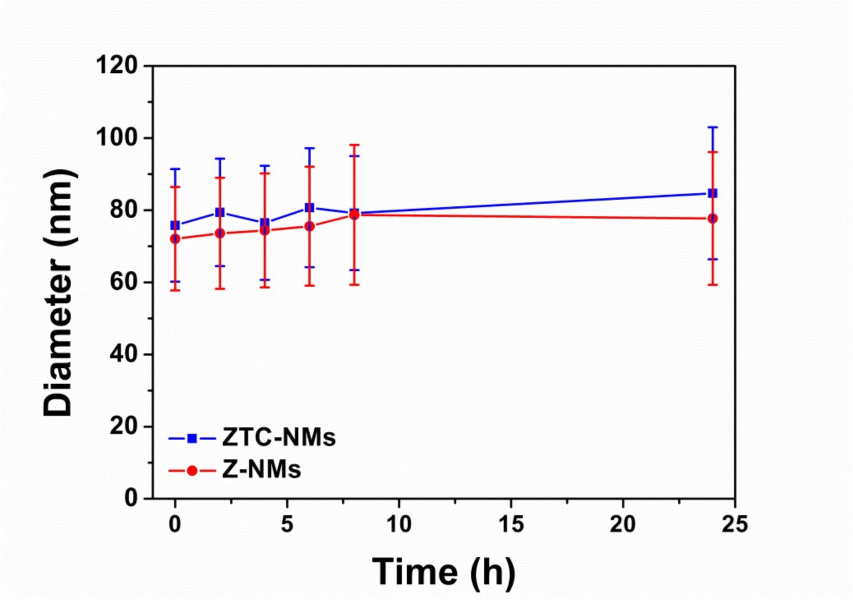

Figure S14. Colloidal stabilities of ZTC-NMs and Z-NMs after incubation with BSA (0.5 mg mL^{-1}).

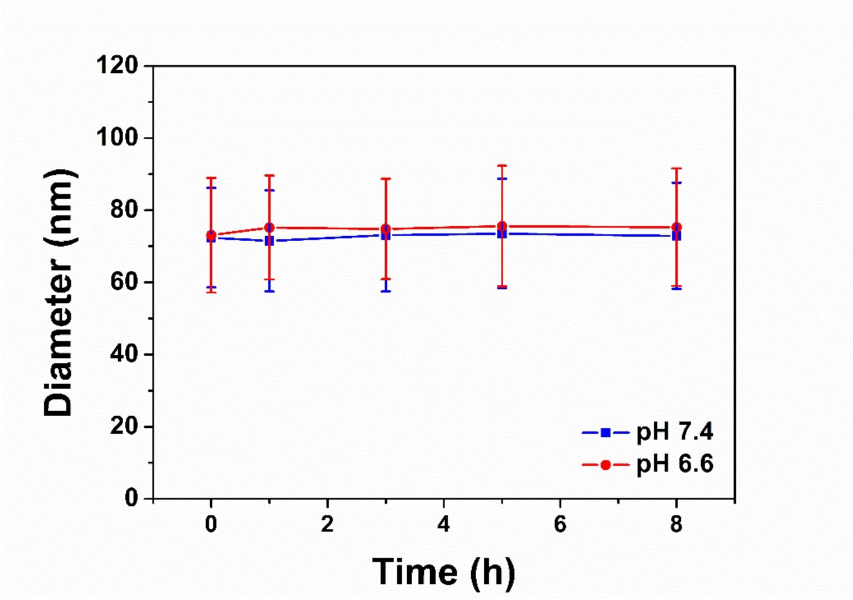

Figure S15. Size changes of ZTC-NMs at different pH values.

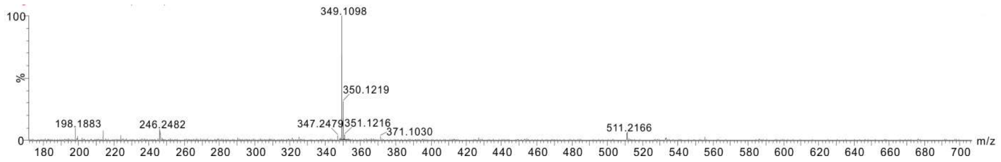

Figure S16. Mass spectrum of the released products from ZTC-NMs with the treatment of 10 mM GSH.

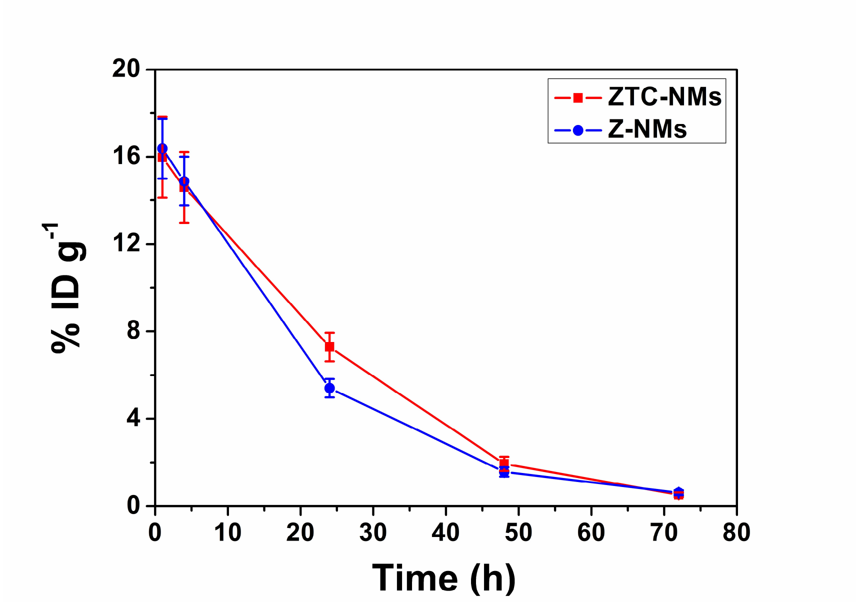

Figure S17. The distribution of Z-NMs and ZTC-NMs in heart (with blood) at different time points after injection.

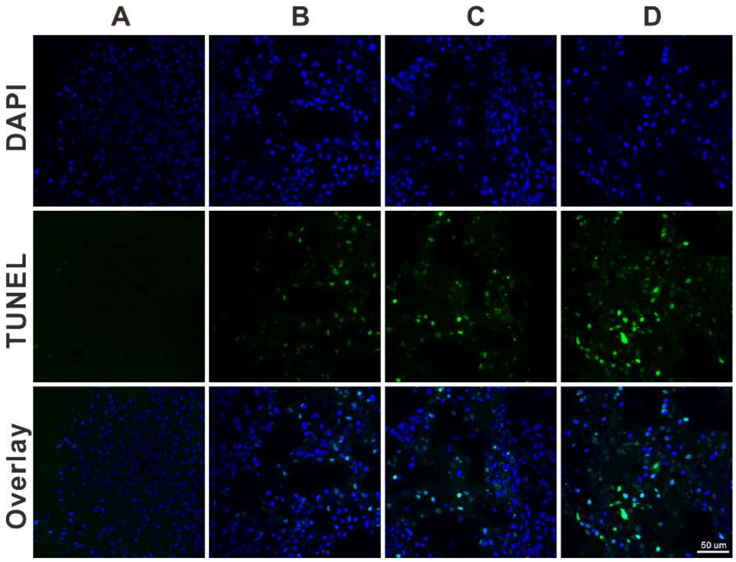

Figure S18. TUNEL analyses of tumor tissues after different treatments. (A) control, (B) free CPT, (C) Z-NMs, (D) ZTC-NMs.

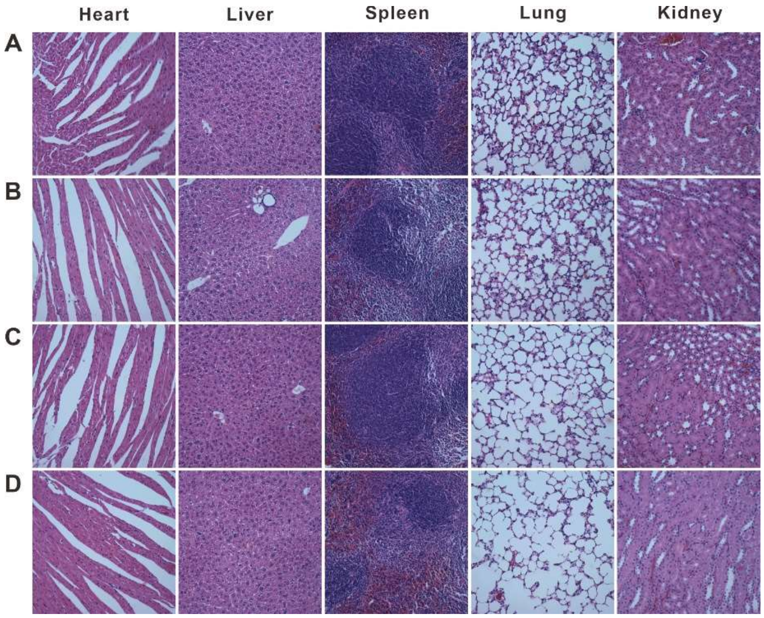

Figure S19. H&E staining of major organs (heart, liver, spleen, lung and kidney) from mice with different treatments. (A) control, (B) free CPT, (C) Z-NMs, (D) ZTC-NMs.
